# Root System Traits Contribute to Variability and Plasticity in Response to Phosphorus Fertilization in 2 Field-Grown Sorghum [*Sorghum bicolor* (L.) Moench] Cultivars

**DOI:** 10.34133/plantphenomics.0002

**Published:** 2022-12-15

**Authors:** Michael O. Adu, Paul A. Asare, David O. Yawson, Kwadwo K. Amoah, Kofi Atiah, Matthew K. Duah, Alex Graham

**Affiliations:** ^1^ Department of Crop Science, School of Agriculture, College of Agriculture and Natural Sciences, University of Cape Coast, Cape Coast, Ghana.; ^2^ Centre for Resource Management and Environmental Studies (CERMES), The University of the West Indies, Cave Hill Campus, P.O. Box 64, Bridgetown BB11000, Barbados.; ^3^ Department of Soil Science, School of Agriculture, College of Agriculture and Natural Sciences, University of Cape Coast, Cape Coast, Ghana.

## Abstract

Due to roots’ physical and physiological roles in crop productivity, interest in root system architecture (RSA) and plasticity in responses to abiotic stresses is growing. Sorghum is significant for the food security of millions of people. Phosphorus deficiency is an important limitation of sorghum productivity. There is little information on the RSA-based responses of sorghum to variations in external P supply ([P]_ext_). This study evaluated the phenotypic plasticity and RSA responses to a range of [P]_ext_ in 2 sorghum genotypes. The results showed that both genotypes responded to [P]_ext_ but with significant variations in about 80% of the RSA traits analyzed. Aboveground biomass and most RSA traits increased with increasing [P]_ext_. Plasticity was both genotype- and trait-dependent. For most RSA traits, the white sorghum genotype showed significantly higher plasticity than the red genotype, with the former having about 28.4% higher total plasticity than the former. RSA traits, such as convex area, surface area, total root length, and length diameter ranges, showed sizeable genetic variability. Root biomass had a high degree of plasticity, but root number and angle traits were the leading contributors to variation. The results suggested 2 root trait spectra: root exploration and developmental spectrum, and there was an indication of potential trade-offs among groups of root traits. It is concluded that RSA traits in sorghum contribute to variability and plasticity in response to [P]_ext_. Given that there might be trade-offs among sorghum root traits, it would be instructive to determine the fundamental constraints underlying these trade-offs.

## Introduction

Roots’ biophysical and physiological functions make them critical for understanding and improving crop productivity and adaptation or resilience to abiotic stresses. Root system architecture (RSA), the spatial arrangement of the root system, mediates root–environment interactions. The plasticity and responses of root systems to environmental conditions, such as nutrients, water, and pH, have been reported [1–3]. Plasticity, or phenotypic plasticity, is often used broadly to refer to the variability of a trait in response to environmental stimuli or the capacity of organisms to alter their phenotype in response to varying environmental conditions [4,5]. While genetic understanding of root system plasticity is evolving, there have been advances in the observational capability of phenotypic modifications in RSA traits in response to environmental conditions. These advances suggest a need for further understanding of RSA traits’ behavior and plasticity for crop resilience under abiotic stress conditions to support crop improvement. Since plasticity might be an adaptive [6] or acclimatization strategy [7], root traits that show plastic responses must be known. As a result, further work is needed on characterizing RSA responses to environmental stimuli, particularly regarding the degree of plasticity of the traits involved [4].

Sorghum (*Sorghum bicolor* L. Moench) is an essential cereal grown globally as a grain, forage, syrup, and energy crop. It is the fifth most important staple food and fodder cereal [8]. Compared to other grain crops, sorghum is abiotic stress-tolerant, particularly for heat and drought, and responsive to more favorable conditions [9]. Sorghums are also relatively better adapted to poor soil fertility and are much more tolerant of low phosphorus (P) levels than wheat or barley [10,11]. The crop is thus popular in semiarid, subtropical, and tropical regions, typical of low-input agricultural systems. In the savannah zones of Africa, where harsh environmental conditions combine with low external nutrient inputs [12], sorghum and pearl millet remain staple food crops [13]. Yet, abiotic stresses, including nutrient deficits, significantly limit the growth and productivity of sorghum [8] and account for the significant yield gaps observed [7].

Phosphorus is a macronutrient that is limiting in most agroecosystems. Among several adaptations, plants are known to change their RSA in response to P concentrations in soils. These changes can increase soil P or enhance P acquisition and fertilizer use efficiency [14]. The plasticity of RSA in response to fluctuating soil resource status provides opportunities to identify valuable root features for efficient soil resource acquisition, especially under deficit conditions [15]. Sorghum has a high P requirement, and low soil P is one of the principal abiotic stresses limiting its production, especially in Africa [13,16,17]. Thus, understanding RSA and the plasticity of the sorghum root system to varied soil P supply can be instrumental in identifying genotypes that use soil P more efficiently and require less fertilizer [8].

Root system traits might also differ among genotypes of a crop species, but genotypic variation in RSA traits, compared to shoot features, has been less explored [18] in sorghum. Genetic variation can be a crucial driver of intraspecific trait variation [19]. Genetic variation contributes to plasticity and provides adaptive or acclimatization options for crops under stressful conditions. Even so, plant breeders have yet to fully explain the extent to which trait variation is due to genotypic or environmental effects (plasticity). Consequently, the relative importance of trait plasticity versus genetic variation, the relationship between the two and the magnitude of plasticity in RSA traits to various responses are often ignored in the breeding process [19]. In cassava, Adu [20] suggested that root features contributing more to variability might also have more remarkable plasticity in response to soil moisture deficit. However, not all influential traits to variability showed plastic responses.

The functional interpretation of root trait plasticity and variability requires the phenotyping of several root system traits concerning plant performance. Adu et al. [21,22] noted that root traits that contribute most to the phenotypic variation among genotypes had not been found for many crop plants. They also stated that trade-offs or redundancies might be in the different root features extracted from root images. Functional root features can thus be classified as groupings of covarying trait suites that reflect adaptive or ecological trade-offs. Traits that differ independently are likely to determine a unique adaptive process. Therefore, elucidating the critical dimensions of functional trait variation is a prerequisite for functional trait-based breeding strategies [23]. The current study was, therefore, designed to answer the following questions: (a) Which root traits of sorghum adapt under soil P limitation? (b) What is the extent of plasticity in root traits in response to P supply? (c) Does the extent of root trait plasticity vary under different rates of P supply? (d) What is the most critical root trait spectrum that contributes to a genotypic variation on sorghum?

## Materials and Methods

### Environmental conditions, soil, and plant material

Two field experiments were done at the Teaching and Research Farms of the School of Agriculture of the University of Cape Coast, Ghana. The study area is located in the coastal savannah agroecological zone. The region has 2 growing seasons, a major season and a minor one, with 100 to 110 and 60 to 70 crop growing days in March to July and September to October, respectively. The dry or harmattan season lasts from November to February. Temperature and relative humidity across the year range between approximately 24 °C and 32 °C and between 60% and 80%, respectively, and the mean annual rainfall was 800 mm. Before fertilization and sowing, composite soil samples were obtained from each field to a depth of 20 cm to determine the chemical properties of the soil. Although the root system of the sorghum plant spreads to at least 1.5 m around the plant and is densest in the top 90 cm of the soil [24], we consider the 20-cm sampling depth informative enough to determine soil chemical properties. The sampling depth for most soils is typically 15 cm because most root activity and fertilizer applications are restricted to this depth. Accordingly, the 0- to 15-cm depth is commonly used for conventional tests of organic matter, P, K, pH, and salt levels. Deeper samples for a deep root crop like sorghum might have been suitable if N fertilizer was the focus [25]. The soil of the experimental site was a haplic acrisol with a pH of 5.5 to 6.1, 2.45% organic carbon, 0.12% total nitrogen (N), 58 μg P g^−1^, and 0.1 cmolc kg^−1^ K.

The genetic materials were obtained from sorghum farmers in the Upper West Region of Ghana. The genetic materials used were 2 commonly grown sorghum genotypes distinguished by the grain pericarp. These were high-yielding short-season varieties, called Naga red and Naga white (hereafter called red and white sorghum), preferred by the locals in the preparation of local beer and food, respectively [26,27]. The varieties were developed through mass selection from a Ghana germplasm collection [27], likely including the red and white sorghum landraces called *Kazie* and *Kapiela*, respectively, by the local farmers [28]. Farmers are shunning these varieties for several reasons, principally due to their need for fertile soils or intensive fertilization to attain higher yields [27].

### Experimental design and field establishment

Between April and August 2020, we established 2 fields under rain-fed conditions. Cassava was the preceding crop, but the fields had been left fallow for a year. The 2 fields were planted 1 month apart and spatially separated by a 100-m fallow. Each trial was set up in a randomized complete block design (RCBD) with 2 blocks and 3 replicated plots. The experimental variables were 2 sorghum genotypes and 6 levels of P supply. The P application rates were chosen to be as close as possible to the fertilizer recommendation rates for sorghum for the present soil. A range of 10.9 to 21.4 mg kg^−1^ soil Olsen-P has been proposed as a critical level for optimal crop yield in different soils [29]. If the P recommendation for sorghum is met, the sufficiency range from seedling to grain filling should range between 0.2% and 0.5% dry matter [30]. Sorghum fertilizer recommendations to achieve this target are not readily available for Ghana’s agroecology and soils. However, 35 to 100 kg ha^−1^ has been proposed for di-ammonium phosphate for all agroecological zones in neighboring west African countries such as Burkina Faso [31]. Accordingly, the 6 P fertilizer rates settled for the present study included 0, 15, 30, 45, 60, and 75 kg P ha^-1^. On each field, the plots consisted of three 5-m rows per genotype per P treatment. Seeds were sown in a nonridged experimental field, which had been ploughed and harrowed to approximately 30 cm. In each field of the experiment, sorghum was seeded manually with a long wooden seed dibber to a depth of 5 to 8 cm and an interrow spacing of 76 cm. Triple superphosphate [TSP, Ca(H_2_PO_4_)2·H_2_O] P fertilizers were used, and no additional or basal fertilizer was applied. Recommended agronomic crop production practices were followed after germination.

### Excavation of root crowns

**Fig. 1.**
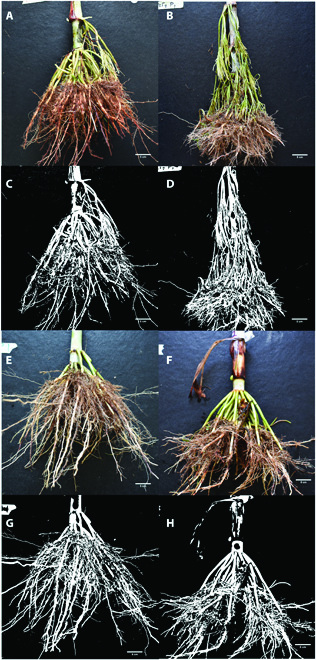
Variation in root system architecture in 2 contrasting sorghum genotypes grown under field conditions. The red genotype is shown in the top panel (A and B), and the white genotype is in the middle panel (E and F). Panels (C) and (D) and (G) and (H) show correspondent segmented images used in extracting root features.

Excavation of root crowns followed the processes described by York [32] for maize. We excavated the root crowns at anthesis, which occurred around 70 days after planting for both genotypes. We dug out 6 plants per plot using a standard shovel. To do so, we firstly made a circle of radius 20 cm around the stem, and along this circle, we pushed the shovel into the soil to a depth of about 30 cm, prying up the root crown slightly on each insertion. At the last insertion, we fully pried up the root crown with the shovel, careful not to disrupt the soil cylinder adhered to the excavated root while lifting it into a basin. We tagged the crowns and carted them to a washing station in a wheelbarrow. We soaked them in water for approximately 60 min. The crowns were then carefully removed and sprayed with clean tap water from the nozzle of a pressurized hose. We gently shook the root crowns, moved them into a large basin of clean water, and brushed and rinsed the roots of remaining soil particles and other debris. We subsequently took images of the roots. Initial observation suggested that some of the root crowns were asymmetric, particularly with uneven development of brace roots around aboveground whorls (Fig. 1). We, therefore, took images from 3 perspectives of each root crown and treated the additional pictures as pseudoreplicates. We placed the cleaned roots and a scale object on a black matte background to take the images and used a Canon EOS 70D DSLR camera, suspended with a tripod 0.6 m above the roots, for the imaging.

### Extraction of root traits

Two data types were extracted from the excavated roots: manual measurements taken directly on the roots and data determined from the root images using image analysis software. Two shoot traits (shoot fresh and dry weight) and 14 root traits were extracted for the manual measures. Biomass traits included root fresh weight (RFW) and root dry weight. Dry biomass was obtained by oven-drying fresh biomass at 70 °C for 72 h. The root traits included the number of whorls (NoW), number of brace roots (NBR), brace root length, branching density of brace roots (BDBr), first arm brace root angle from first and second whorls in relation to horizontal (BA1a/b), and second arm brace root angle from first and second whorls in regard to horizontal (BA2a/b). Other root traits extracted manually were the number of crown roots, crown loot length, branching density of crown roots, and angle of crown roots. The branching density of each root type was calculated within 3 to 5 cm and 1 cm of the root’s origin, and we assumed that the branching density would remain constant over the rest of the root’s axis. The root angles were measured with the “shovelomics scoreboard” developed by Trachsel et al. [33].

We used the “Whole Root” mode in the Rhizo Vision Explorer, version 2.0.3 [31], to analyze the root images to extract RSA features of intact sorghum root crowns. The options for image thresholding level, edge smoothing, and root pruning threshold were set at 230-pixel intensity, 2, and 1 pixel, respectively. The pixels for diameter ranges 1, 2, and 3 were 0 to 2, 2 to 5, and above 5, respectively. Pixels were subsequently converted to SI units (mm). The RhizoVision Explorer, increasingly gaining popularity among root “phenotypers”, is an innovative free and open-source software for estimating root features from images acquired by scanners or cameras [34]. The software enables the extraction of root features that cannot be determined manually. These root features include average hole size (AHS), average root orientation, convex area, depth, holes, lower root area, maximum diameter, the maximum number of roots, maximum width, median diameter, the median number of roots, medium angle frequency, and network area. The Table defines the abbreviations for all traits extracted for this study, including the 37 attributes extracted from root images. The descriptions of the novel features extracted from the root images are provided by Seethepalli et al. [35,36].

**Table.** Means, standard deviations, ranges, coefficient of variation, variance, and broad-sense heritability estimates for shoot and root system traits of 2 genotypes of sorghum grown under field conditions. Ranges for features with multiple categories are as follows: range 1: 0 to 2 mm, range 2: 2 to 5 mm, and range 3: >5 mm.

Root system trait (unit)	Acronym	Root trait group	Summary statistics				ANOVA			*H* ^2^
			Mean	SD	Range	CoV	F prob. Geno	F prob. P_[ext]_	F prob. Geno × P_[ext]_	
Angle of crown roots (°)	CRA	Angle	57.0	90.0	17.8	31.3	0.968	<0.001	0.006	-
Average diameter (mm)	AvD	Diameter	8.4	7.2	0.6	7.7	0.084	0.023	0.025	-
Average hole size (mm^2^)	AHS	Area	127.2	543.1	49.7	39.1	<0.001	0.002	0.338	0.28
Average root orientation (°)	ARO	Angle	48.6	18.3	2.4	4.9	<0.001	0.003	0.481	-
Brace root length (cm)	BRL	Length	17.2	30.0	4.0	23.5	<0.001	0.189	<0.001	-
First arm brace root angle first whorl (°)	BA1a	Angle	107.2	180.0	37.5	34.9	0.605	0.004	0.005	-
First arm brace root angle second whorl (°)	BA1b	Angle	107.8	182.0	42.6	39.5	0.04	<0.001	0.01	0.02
Second arm brace root angle first whorl (°)	BA2a	Angle	113.8	180.0	38.5	33.8	<0.001	0.352	0.116	0.54
Second arm brace root angle second whorl (°)	BA2b	Angle	105.4	175.0	44.0	41.7	0.241	0.26	0.016	-
Branching of brace roots (root no./cm)	BDBr	Number	5.2	21.0	3.5	67.2	0.038	<0.001	0.009	0.04
Branching of crown roots (root no./cm)	BDCr	Number	5.7	14.0	2.8	50.1	0.333	0.017	0.155	-
Convex area (mm^2^)	CoA	Area	389,247	1,140,215	176,864	45.4	<0.001	0.006	0.357	1.00
Crown root length (cm)	CRL	Length	17.5	29.3	3.7	21.4	<0.001	0.003	0.002	-
Depth (mm)	Dep	Length	821.0	702.2	128.8	15.7	<0.001	0.286	0.639	-
Holes	Hol	Number	427.8	1,566.0	223.1	52.2	<0.001	<0.001	0.003	0.58
Lower root area (mm^2^)	LRA	Area	115,848	381,202.0	57,533	49.7	<0.001	0.462	0.102	0.98
Maximum diameter (mm)	MxD	Diameter	35.7	37.0	5.8	16.3	0.024	0.156	0.265	-
Maximum number of roots	MxNR	Number	30.3	53.0	7.9	26.0	<0.001	0.014	0.028	-
Maximum width (mm)	MW	Width	708.5	1,111.0	222.1	31.3	<0.001	0.008	0.232	0.42
Median diameter (mm)	MeD	Diameter	7.5	9.6	0.7	9.4	<0.001	0.151	0.007	-
Median number of roots	MNR	Number	10.0	26.0	4.3	43.0	<0.001	0.007	0.015	0.54
Medium angle frequency	MAF	Number	0.3	0.2	0.0	8.1	<0.001	0.465	0.795	-
Network area (mm^2^)	NeA	Area	134,593	397,535.0	65,490	48.7	<0.001	0.012	0.002	0.22
Number of crown roots	NCR	Number	4.2	9.0	1.8	41.4	0.935	<0.001	<0.001	-
Number of root tips	NRT	Number	514.3	1,412.0	276.4	53.7	<0.001	<0.001	0.018	0.62
Number of brace roots	NBR	Number	34.0	84.0	13.1	38.3	<0.001	<0.001	<0.001	0.18
Numbers of aboveground whorls	NoW	Number	3.3	8.0	1.2	35.4	<0.001	0.001	0.03	0.04
Perimeter (mm)	Per	Length	29,021	72,219.0	13,029	44.9	<0.001	0.004	0.02	0.07
Projected area diameter—range 1 (mm^2^)	PAD1	Area	977.5	2,693.0	509.2	52.1	<0.001	<0.001	0.084	0.40
Projected area diameter—range 2 (mm^2^)	PAD2	Area	20,349	48,596.0	8,578.0	42.2	<0.001	<0.001	0.155	0.44
Projected area diameter—range 3 (mm^2^)	PAD3	Area	181,655	619,355.0	94,510	52.0	<0.001	0.01	0.001	0.55
Root dry weight (g)	RDW	Biomass	78.2	437.5	88.9	113.7	0.104	0.005	0.589	-
Root fresh weight (g)	RFW	Biomass	189.0	1,235.0	244.9	129.6	<0.001	0.003	0.193	0.89
Root length diameter—range 1 (mm)	RLD1	Length	720.8	2,007.0	378.3	52.5	<0.001	<0.001	0.08	0.37
Root length diameter—range 2 (mm)	RLD2	Length	5,373.0	12,899.0	2,287.0	42.6	<0.001	<0.001	0.173	0.43
Root length diameter—range 3 (mm)	RLD3	Length	17,965	56,438.0	9,004.0	50.1	<0.001	0.007	0.002	0.98
Shallow angle frequency	ShAF	Number	0.3	0.3	0.0	10.2	<0.001	0.002	0.126	-
Shoot dry weight (g)	SDW	Biomass	196.0	905.8	153.7	78.4	0.221	<0.001	<0.001	-
Shoot fresh weight (g)	SFW	Biomass	333.7	1,174.0	260.9	78.2	0.929	0.29	0.021	-
Solidity	Sol	Area	0.3	0.4	0.1	20.2	0.426	<0.001	0.074	-
Steep angle frequency	StAF	Number	0.4	0.3	0.0	10.9	<0.001	0.009	0.784	-
Surface area diameter—range 1 (mm)	SAD1	Diameter	3,071.0	8,459.0	1,600.0	52.1	<0.001	<0.001	0.084	0.40
Surface area diameter—range 2 (mm)	SAD2	Diameter	63,929	152,670.0	26,947	42.2	<0.001	<0.001	0.155	0.44
Surface area diameter—range 2 (mm)	SAD3	Diameter	570,708	1,945,819	296,924	52.0	<0.001	0.01	0.001	0.23
Surface area (mm^2^)	SuA	Area	637,708	2,067,990	321,385	50.4	<0.001	0.007	0.001	0.77
Total root length (mm)	TRL	Length	24,059	67,412.0	11,438	47.5	<0.001	0.003	0.006	0.47
Volume diameter—range 1 (mm^3^)	VDR1	Volume	1,140.0	3,154.0	590.0	51.8	<0.001	<0.001	0.09	0.40
Volume diameter—range 2 (mm^3^)	VDR2	Volume	63,377	150,717.0	26,524	41.9	<0.001	<0.001	0.14	0.41
Volume diameter—range 3 (mm^3^)	VDR3	Volume	1,717,684	6,161,094	911,057	53.0	<0.001	0.01	0.001	0.62
Volume (mm^3^)	Vol	Volume	1,782,201	6,280,013	932,323	52.3	<0.001	0.009	0.001	0.62
Width-to-depth ratio	WDR	Width	0.9	1.6	0.2	25.4	<0.001	0.169	0.029	-

### Data analysis

We employed various processes to progressively reduce the dimensions of the 51 traits measured in this study. We initially eliminated features based on the magnitude of the coefficient of variation (CoV) or differences between genotypes [22]. First, we combined the data from the 2 trials and obtained summary statistics such as mean (*x*), standard deviation (*σ*), range, and CoV. For further analysis, we maintained traits with CoV values ≥ 30% [37]. Subsequently, we performed a general analysis of variance to determine differences in measured traits between the 2 genotypes. We excluded any traits that presented a nonsignificant difference between genotypes [22]. We then estimated broad-sense heritability (*H*^2^) across the experiments based on variance components for the remaining traits, employing residual maximum likelihood (REML) procedures. We categorized all factors as random in the REML analyses so that the proportional contribution of genotype to overall variation in traits could be determined [21]. The random model (Eq. 1) for the REML analyses was:

$$\begin{array}{c} y_{\text{ijkl}}\;=\;\mu +g_{i}\;+\;p_{j}\;+\;{\mathrm{gp}}_{\mathrm{ij}}+{\mathrm{pr}}_{\mathrm{jk}}+{\mathrm{gpr}}_{\mathrm{ijk}}+{\mathrm{prb}}_{\mathrm{jkl}}+{\text{gprb}}_{\text{ijkl}}+\varepsilon_{\text{ijkl}} \end{array}$$ (1)

where *y*_*ijkl*_ represents the observation from the *ijkl^th^* genotype, P supply rate, experimental run, and block; *μ* is the overall mean; *g_i_* is the effect of the *i^th^* genotype; *p_j_* is the effect of the *j^th^* P rate; *gp*_*ij*_ is the interactive effect of the *i^th^* genotype with the *j^th^* P supply rate; *pr*_*jk*_ is the interactive effect of the *j^th^* P supply rate with the *k^th^* experimental run; *gpr*_*ijk*_ is the interactive effect of the *i^th^* genotype with the *j^th^* P supply rate and the *k^th^* experimental run; *prb*_*jkl*_ is the interactive effect of the *j^th^* P supply rate, the *k^th^* experimental run, and the *l^th^* block; *gprb*_*ijkl*_ is the interactive effect of the *i^th^* genotype with the *j^th^* P supply rate, the *k^th^* experimental run, and the *l^th^* block; and *ε*_*ijkl*_ is the experimental error.

We estimated *H*^2^ as the quotient of the genotypic variance and the total phenotypic variance for the trait (*σ_g_*^2^/*σ_p_*^2^) [38]. We employed Eq. 2, as applied by [22], to estimate the phenotypic variance, where *r* is the number of replicates, *n* is the number of trials, and *σg*^2^ × *t* is the genotype× trial variance.

$$\sigma_{p}^{2}=\frac{\sigma_{g}^{2}\times t}{n}+\frac{\sigma_{\varepsilon }^{2}}{\mathrm{rn}}$$ (2)

We used multiple factor analysis (MFA), a multivariate ordination procedure, to identify significant root trait groupings accounting for most variation among the genotypes and provide an integrated picture of the root traits and the relationships between trait groupings. Thus, the MFA tested if root trait groupings covaried. Generally, MFA searches for similarities in datasets by doing a principal component analysis (PCA) on each data. The data are normalized as the quotient of its elements and the square root of the first eigenvalue from its PCA. In the second step of the MFA, the normalized datasets are combined to form a distinctive matrix on which a global PCA is performed. Individual datasets are projected onto a global analysis to uncover communalities and differences [39]. Unlike PCA, MFA organizes traits in groups and finds connections based on RV and Lg coefficients. The RV, which ranges between 0 and 1, computes the similarity coefficients and reflects correlation or the amount of variance shared by 2 groups of traits, with coefficients approaching 1 indicating stronger relationships [40]. The Lg coefficient is a dimensionality indicator that evaluates the relationship between groups of variables. In this study, the MFA summarized the variables into 2 categorical variables (genotype and P supply) and 8 root trait groupings. The genotype and P supply groups specified identity and external P fertilizer supply rates. For each genotype and P supply, the value of each trait grouping was the mean value for all replications. The remaining groups were continuous variables. We based the factorability of the root traits on well-recognized criteria for describing root systems in terms of traits that measure geometry, morphology, topology, and growth dynamics [41]. Accordingly, we factorized traits into root angle, area, biomass or weight, diameter, length, volume, and width.

We employed the relative distance plasticity index method (RDPI) proposed by Valladares et al. [5] to determine the plasticity of sorghum root traits under different external P supply rates. We assessed root phenotypic plasticity for all features having CoV values ≥ 30% and for which there was a significant difference between genotypes. We adapted the procedure of Lak et al. [42] to calculate RDPI. The RDPI ranges from 0 (no plasticity) to 1 (maximum plasticity) [20,43,44]. In this study, the RDPI specifies the relative phenotypic distance or amount of change in a given trait between plants of the same genotype exposed to different rates of P fertilization. For each trait (*x*), RDPI_(*x*)_ values were determined by dividing the sum (*x_i′j′_* + *x_ij_*) by the relative phenotypic distances across replicates (*d_ij_*→*i_’j’_*) of the same genotype under varied P supplies. For each genotype, an RDPI ranging from 0 to 1 was calculated as follows:

$$\begin{array}{c} \text{RDPI}=\sum \left(d_{\mathrm{ij}}\to i_{\prime j^{\prime }}/\left(x_{i^{\prime }j^{\prime }}+x_{\mathrm{ij}}\right)+n\right) \end{array}$$ (3)

where *n* is the total number of distances. Adapting the procedure of Lak et al. [42], we computed 6 types of RDPIs: (a) RDPI_Total,_ considering relative phenotypic distances between all P levels; (b) RDPI_P15_ vs. P_0_ (P_15_), weighing relative phenotypic distances between traits under 15 and 0 kg P/ha only; (c) RDPI_P30_ vs. P_0_ (P_30_), considering relative phenotypic distances between root traits under 30 and 0 kg P/ha only; (d) RDPI_P45_ vs. P_0_ (P_45_), weighing relative phenotypic distances between root features under 45 and 0 kg P/ha only; (e) RDPI_P60_ vs. P_0_ (P_60_), considering relative phenotypic distances between traits under 60 and 0 kg P/ha only; and (f) RDPI_P75_ vs. P_0_ (P_75_), weighing relative phenotypic distances between root features under 75 and 0 kg P/ha only.

We performed REML and ANOVA analyses with GenStat (GenStat Release 12.1, VSN International, Oxford, UK). We used the FactoMineR package in the R software, the Language and Environment for Statistical Computing [45,46] for the MFA, and the package Factoextra to visualize the MFA results [45]. We calculated the RDPI values with the “Plasticity” package [47] implemented in the R software [46].

## Results

### Measures of variability differed among traits

We obtained descriptive statistics for 51 traits consisting of 2 shoot traits and 49 root traits (Table). There was an approximately 2-fold difference between the crown roots angle and the first whorl’s second arm brace root angle (Table). Between the 3 projected area diameters (ranges 1 to 3), range 1 recorded the least mean area and range 3 recorded the highest, with about a 186-fold difference between the two (Table). The trend was similar for the surface area diameters, root length, and volume diameter ranges (Table). Shoot biomass was more significant than root biomass, but brace root and crown length were nearly equal (Table). The CoVs for the traits ranged from 4.7% (average root orientation) to 129.6% (fresh root weight). The CoV was largest (≥60%) for branching of brace roots, shoot, and root biomass traits, and intermediate (30% to 59%) for 33 features, including angles of the crown and some brace roots, number of brace roots and aboveground whorls, perimeter, total root length, convex, and network area. The CoV was smallest (<0.30) for 13 traits eliminated from the subsequent multivariate analyses. These traits included average root orientation, median, maximum diameters, angle frequencies, depth, and solidity ( Table).

### Significant differences among the genotypes were found in most traits

**Fig. 2.**
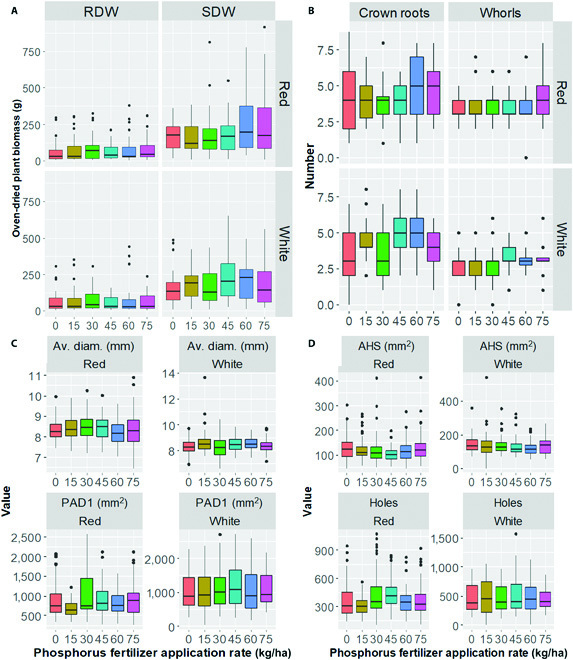
Box plots of the root architecture traits for 2 sorghum genotypes (white and red pericarp genotypes) grown at 6 P availability. (A) Shoot and root biomass. (B) Number of crown roots and aboveground whorls. (C) Average diameter and projected area diameter—range 1. (D) Number of holes and average hole size. Acronyms for variables are defined in the Table.

Variation in the root architecture of the 2 genotypes is presented in Figs. 1 and 2. There were significant differences among the genotypes (*P* ≤ 0.001 or *P* ≤ 0.05) for approximately 80% of the traits evaluated (Table), and for these traits, the white sorghum recorded greater values in 79%. The red genotype had more shoot and root biomass (Figs, 1 and 2A and 2B) and greater aboveground whorls than the white genotype (Figs. 1 and 2B). The white genotype was higher in average diameter, projected area diameter (Fig. 2C), and number and size of holes (Fig. 2D). In traits for which the white genotypes were superior, the genetic variability between the 2 genotypes ranged from 2.5% (medium angle frequency) to 23.5% (root length diameter range 3). Where the red genotype presented bigger values, the genetic variability between the 2 genotypes ranged from 1.6% (average root orientation) to 31% (fresh root weight).

We found significant differences (*P* ≤ 0.001 or *P* ≤ 0.05) among the P fertilizer rates in 82% of the traits evaluated (Table). There was increasing plant biomass with increasing external P concentration, but biomass declined from 60 kg P ha^−1^ (Fig. 2A). The effect of P on the root traits did not present consistent trends for the 2 genotypes (Figs. 2B to 2D). Both projected area diameter (range 1) and average diameter increase with increasing P and plateau or decline (Fig. 2B). The most significant number of whorls and crown roots were recorded at higher rates of P supply for both genotypes (Fig. 2D), but the reverse seems valid for the average hole size (Fig. 2C). Estimates of broad-sense heritability (*H*^2^) were derived from variance components for 30 traits (Table). The *H*^2^ estimates were generally low to high and ranged from 0.02 (first arm brace root angle of the second whorl) to 1.0 (convex area) (Table). The *H*^2^ was large (≥0.60) for 8 traits, including volume, volume diameter—range 3, number of root tips, surface area, and fresh root weight ( Table). The *H*^2^ estimates were intermediate (0.25 to 0.59) for 15 traits, including average hole size, range 1 of root length diameter, volume diameter, projected area diameter, and surface area diameter ( Table). The *H*^2^ estimate was the smallest (<0.25) for traits such as numbers of aboveground whorls and brace roots, branching of brace roots, perimeter, and network area (Table).

### Multiple factor analyses

#### Strong correlations were found among many traits and trait groups

**Fig. 3.**
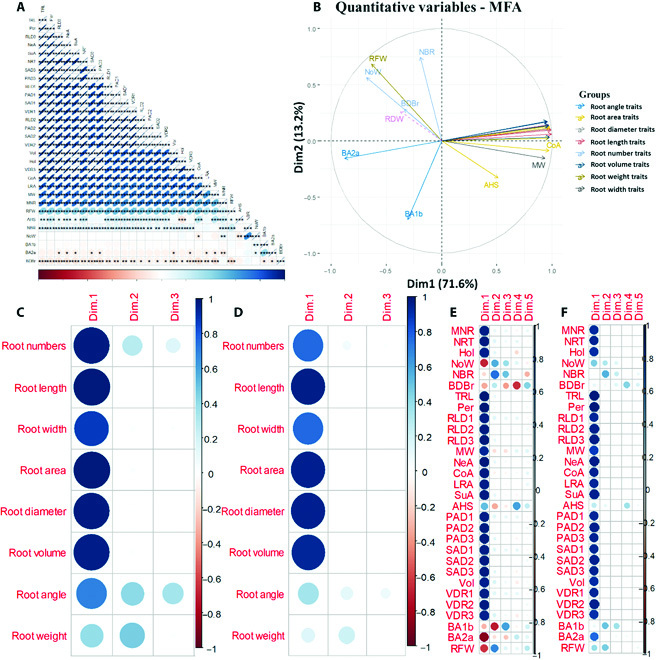
Results of correlation and multiple factor analysis (MFA). (A) Correlations between plant traits. The scale of color codes and eccentricity of the ellipses indicate the correlation coefficients between the 2 traits. The scale is indicated in the bar below the matrix. Blank boxes indicate nonsignificant relationships and asterisks indicate significance at *P* < 0.05, *P* < 0.01, or *P* < 0.001. (B) Correlation between quantitative variables and dimensions by groups of traits. Overlapping trait names have been removed for clarity. (C) Loading scores of variable groups of the first 3 dimensions, including the 2 significant dimensions. (D) Plot of quality of representation of the variable groups (cos^2^ of variable groups) on the factor map for the first 3 dimensions, including the 2 dimensions considered significant following the MFA. (E) MFA results for individual quantitative variables. (F) cos^2^ of individual quantitative variables. Acronyms for variables are defined in the Table.

There were strong positive correlations among many traits (Fig. 3A). Total root length was strongly positively correlated (*r* = 0.12 to 0.99; *P* < 0.001) with several traits, including perimeter, network area, number of root tips, projected area diameters, surface area diameters, and root length diameters. Fresh root biomass was positively but moderately or weakly correlated (*r* = 0.18 to 0.48; *P* < 0.001) with several traits, including total root length, perimeter, network and convex areas, hole size, and various diameter ranges. There were also some negative but significant correlations (Fig. 3A). Branching of brace roots negatively correlated (*r* = 0.12 to 0.16; *P* < 0.05; 0.01 or 0.001) with traits such as total root length and maximum width. Little correlation was found between the number of aboveground whorls or the root insertion angle traits (BA1a, BA1b, and BA2b) and other traits (Fig. 3A). The correlation circle indicated over 20 positively correlated variables, well represented on the factor map (Fig. 3B). The plot of the groups of variables suggested a strong correlation between trait groups and dimensions. Four variable groups corresponding to 4 positively correlated groups can be identified in the biplot of PC1 and 2 (Fig. 3B). The first group had fewer traits but was dominated by root area traits (AHS and convex area). The second group had 21 positively correlated traits, a mixture of several trait groupings except for root number, angle, and width traits. Groups 1 and 2 negatively correlated with group 3, which was dominated by root number-related features (NoW, NBR, and BDBr), and group 4 traits, which were primarily composed of root insertion angle-related features (Ba1b and BA2a) (Fig. 3B).

The first and second factors had eigenvalues of over one and explained 71.6% and 13.2% of the variance, respectively, providing a 2-factor solution, which explained approximately 85% of the variance (Fig. 3B). In the first dimension, the coordinates of 5 active groups, including trait groups for root length, area, volume, and diameter, were almost identical. In support of this observation, the MFA suggested that root traits representing root length, root width, root area, root diameter, and root volume are close because the RV coefficients were closer to 1 (RV = 0.78 to 1.00). The RV coefficients for the mean configuration of the MFA for groups of traits representing root numbers, root length, root width, root area, root diameter, root volume, root angle, and root weight were 0.92, 0.96, 0.86, 0.97, 0.96, 0.95, 0.67, and 0.48, respectively. Thus, root area traits recorded a closer RV coefficient (0.97) to the mean configuration of the MFA. All the groups on the first dimension of the MFA obtained high Lg values (≥1), with root number traits (Lg = 1.25) and root angle traits (Lg =1.37) recording the highest Lg coefficients. Three active groups (root numbers, angle, and weight) resolved in the second dimension, but root weight had the highest coordinate in this dimension (Fig. 3C). From the estimated squared cosine of a component (cos^2^), all the trait groupings contributed more significant portions to the total distance than amounts contributed by root angle and root biomass groups (Fig. 3D). The quantitative variable coordinates (Fig. 3E) indicated that the variation in the first axes was primarily explained by 23 of the 30 traits factorized for the MFA. The 7 remaining root traits that described the variation on the second dimension include fresh root biomass, the number of aboveground whorls, and brace root-related traits (BDBr, NBR, BA1b, and BA2a) (Fig. 3E). From the cos^2^ values, about 26 root features that resolved on the first dimension contributed relatively largely to the total distance of the observation to the origin (Fig. 3F).

#### Trait groupings contributed to principal components

**Fig. 4.**
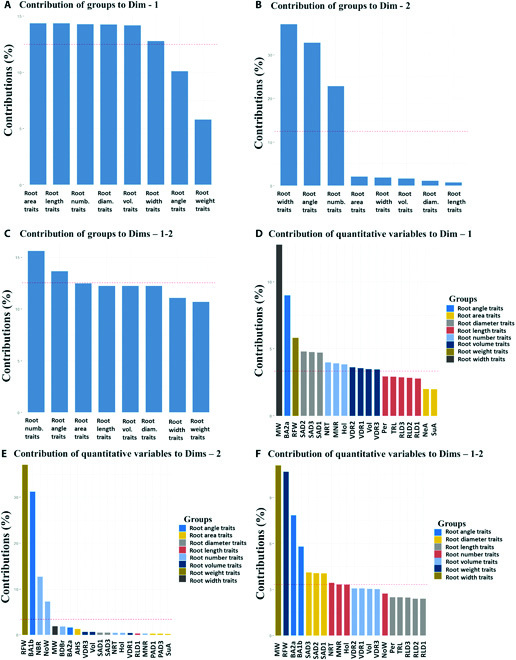
Plot of the contribution of variable groups and individual variables to dimensions from the MFA. (A) Contribution of variable groups to the first dimension. (B) Contribution of variable groups to the second dimension. (C) Contribution of variable groups to the first 2 dimensions. (D) Contribution of individual quantitative variables to the first dimension. (E) Contribution of individual quantitative variables to the second dimension. (F) Contribution of individual quantitative variables to the first 2 dimensions. Acronyms for variables are defined in the Table.

The contributions of variable groups accounting for the variability in the principal component are shown in Figs. 4A to 4C. Six out of the 8 groups correlated with the first, but the coordinates of 5 active groups were almost identical and thus contributed similarly to the first dimension (Fig. 4A). Three of the 8 groups correlated with the second PC (Fig. 4B). Groups associated with PC1 included root area, length, number, diameter, and volume traits (Fig. 4A). Root width and angle traits were among those that correlated with PC2 (Fig. 4B). However, 2 groups (root number and angle trait groups) contributed above the average cutoff point to the variability in the first 2 dimensions (PC1 and PC2) (Fig. 4C). Approximately 43% of the 30 root traits contributed above the average cutoff point to the variability in PC1 (Fig 4D). The most prominent contributing traits were maximum width, second arm brace root angle of the first whorl, and fresh root weight (Fig. 4D). Four individual variables contributed above average to the variability in PC2. They were ranked in the order of fresh root weight, first arm brace root angle of the second whorl, the number of brace roots, and aboveground whorls (Fig. 4E). Eight traits contributed to the variability in the first 2 dimensions (Fig. 4F). The majority of these traits were diameter-related traits (SAD1 to SAD3). Still, the highest contributor traits were root width-related traits (maximum width), root weight-related traits (RFW), and root angle-related traits (BA2a and BA1b) (Fig. 4F).

#### Relative distance plasticity index depended on genotype and measured trait

**Fig. 5.**
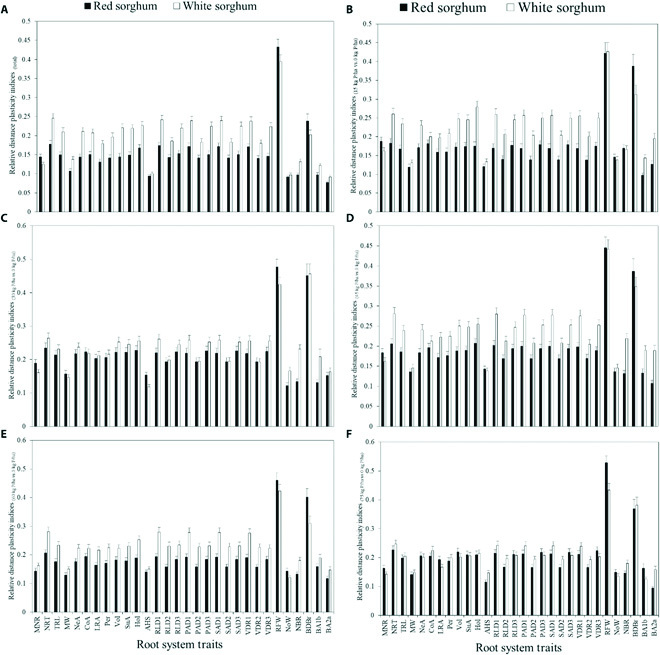
Relative distance plasticity indices (RDPI) for the quantitative root traits of mature field-grown sorghum plants established under varying P supply rates. (A) Relative phenotypic distances (RDPI_Total_) between all P supply levels. (B) RDPI between traits under 15 and 0 kg P/ha only. (C) RDPI between root features under 30 and 0 kg P/ha only. (D) RDPI between traits under 45 and 0 kg P/ha only. (E) RDPI between traits under 60 and 0 kg P/ha only. (F) RDPI between root features under 75 and 0 kg P/ha only. Acronyms for variables are defined in the Table.

The relative distance plasticity indices (RDPIs) showed that plasticity depended on the genotype and measured trait (Fig. 5A). RDPI_Total_ was low (≤0.3) to moderate (0.3 to 0.5), ranging from 0.08 ± 0.004 (BA2a) to 0.433 ± 0.020 (RFW) for the red sorghum and from 0.092 ± 0.004 (BA2a) to 0.394 ± 0.018 (RFW) for the white sorghum. All but one trait (RFW) recorded low indices for RDPI_Total_ (Fig. 5A). RDPI_Total_ for all but 4 traits (AHS, RFW, NoW, and BDBr) of the white sorghum was significantly (*P* < 0.05) greater than those for the red sorghum (Fig. 5A). On average, the RDPI_Total_ of white sorghum was 28.4%, significantly greater (*P* = 0.007) than red sorghum’s. The RDPI for RDPI_P15_ vs. P_0_ ranged from 0.10 ± 0.01 (BA1b) to 0.422 ± 0.03 (RFW) for the red sorghum and from 0.131 ± 0.001 (MW) to 0.427 ± 0.02 (RFW) for the white sorghum (Fig. 5B). For 22 out of the 30 root traits (≈73%), the RDPI_P15_ vs. P_0_ was significantly higher in the white genotype, recording between 1.2% and 59% higher indices than corresponding traits in the red genotype. For RDPI_P30_ vs. P_0_, there was no significant difference between RDPIs of the white and red sorghum for 80% of the root traits included in the analyses (Fig. 5C). Generally, the RDPI for both genotypes showed a consistent comparison trend between 0 kg P ha^−1^ on one side and 45 and 60 kg P ha^−1^ on the other (Figs. 5D and 5E). The white sorghum genotype showed significantly higher plasticity than the red sorghum for all these comparisons of P supply (Figs. 5D and 5E). The average RDPI recorded by the white and red sorghum genotypes were 0.24 and 0.19 (RDPI_P45_ vs. P_0_) and 0.23 and 0.19 (RDPI_P60_ vs. P_0_), respectively. On average, there was no significant difference between the RDPIs of the white and red sorghum for comparing RDPI_P75_ and P_0_ (Fig. 5F). In most of the comparisons, the average hole size of the root system showed the least plasticity index and root fresh weight and branching of brace roots showed the greatest index (Figs. 5A to 5F).

## Discussion

### Significant genetic variations were observed for the majority of the traits

Understanding sorghum RSA traits and plastic responses to variations in soil P can be instrumental to the selection, breeding, and improved productivity of efficient genotypes. In this study, we investigated variations in root traits in 2 sorghum genotypes. The study sought to answer the following questions: (a) Which root traits of sorghum adapt under soil P limitation? (b) What is the extent of plasticity in root traits in response to P supply? (c) Does the extent of root trait plasticity vary under different rates of P supply? (d) What is the most critical root trait spectrum that contributes to a genotypic variation on sorghum? No root system genetic data for the 2 species used in this study are available. Still, root trait values revealed significant variation between the genotypes, suggesting potential genetic differences. The white genotype showed superiority in many traits, suggesting a possible systematic pattern that could hierarchize the genotypes, but this hierarchy cannot be immediately explained. The 49 root traits investigated form valuable descriptors of the RSA and its variability and can thus summarize roots’ developmental processes and characteristics [48].

Significant genetic variations were observed for the majority of the traits. Still, the magnitude of variations differed across the root traits, similar to the findings of Beroueg et al. [48] for *Lactuca* genotypes. Given that the extent of genetic variability for a given trait signifies its improvement, the observation here suggests that there are divergent prospects for improving the features measured in this study. Root system traits that showed sizeable genetic variabilities, such as convex area, surface area, total root length, volume, and root length diameter ranges, could be easy targets for improvements through breeding. Conversely, traits that showed low genetic variabilities, such as BDBr, average root orientation, median diameter, and medium and shallow angle frequency, might be intractable when breeding for improvement to adapt to environmental conditions [49]. The variation observed under varying or natural environments would be explained simply by the plastic response to different environments for traits that showed low or no genetic variation under controlled environmental conditions.

CoVs for the measured root traits varied considerably (Table), and the observed range (4.7% to 129.6%) in the current study is higher than that reported for root traits in oilseed rape (*Brassica rapa*) seedlings (5.8% to 83.2%) [38]. The extensive range of CoV holds for root traits for which intrinsic noise, or developmental stochasticity, is particularly significant [38]. The fact that the larger CoVs were mainly associated with biomass traits ( Table) points to the possible influence of trait dimension on the magnitude of the CoVs. The higher CoV for fresh root weight than for dry root weight implies that fresh weight was more variable, suggesting that dry weights provided a more precise measurement of biomass than fresh weight. Bashan and de-Bashan [50] observed similarly and noted that fresh weight determinations might be altered by factors independent of the intended experimental variables. Pélabon et al. [51] argued that CoVs differ according to the number of dimensions of the measurement, where length, area, and volume consist of 1, 2, and 3 dimensions, respectively. For example, the CoV of a root volume will be 3 times the CoV for root length. The higher CoVs observed here for biomass were, therefore, unsurprising. Even so, it might be critical to characterize developmental stochasticity to minimize residual variations in root biomass traits during phenotyping experiments, especially in uncontrolled or field environments. When CoV values for given attributes are low to moderate, it indicates that variations observed in the characteristic between treatments are likely due to the effect of the intended experimental variables but not factors independent of it [52]. Thus, the current study’s differences in biomass traits due to genotype or external P supply are relatively minimal. Root mass measurements are expected to have larger CoVs than root length or area measurements. Similar to the report of Adu et al. [22] for field-grown cowpea, broad-sense heritability was high for root biomass (0.89). High heritability could indicate a minimal environmental influence on a trait [22]. Therefore, the high heritability recorded for root biomass nullifies the effect of vagaries in experimental conditions on its CoV and might give credence to dimensionality’s influence on its magnitude.

### Both the sorghum and the soil used responded to external P supply

In the present study, the shoot biomass of the sorghum genotypes increased to an asymptote with increasing P addition to the soil (Fig. 2A). This demonstrates that both the sorghum and soil responded to external P. This soil will thus need P additions to achieve growth approaching the physiological maximum for the crop [53]. Some reports suggest that sorghum thrives in low-P conditions by stabilizing the number and length of its lateral roots but with a slight penalty in growth [8]. Most root traits, particularly root diameter traits, increase in response to abiotic stress conditions [54]. When cultivated under P deprivation, most species partition a more significant proportion of their total dry matter into root development [55]. The average hole size (Fig. 2C), which might indicate the degree of root branching and complexity [34], increased with decreasing P supply in the present study. However, most root traits increased to an asymptote with increasing P application (Fig. 2B). This inconsistency may be associated with either the difference in species with unique response mechanisms to P availability or differences in place of the evolution of plant species or the growth stage of assessing root traits. Moreover, we do not have complete data on the availability of all macro- and micronutrient in the soil used for the present study, but the total N in the soil was 0.12%, which is low and might be insufficient to meet the crop’s demand [56]. Therefore, it is possible that plants grown in high-P conditions were no longer in a deficit of P but were still foraging for other nutrients, effectively confounding the manifestation of the typical root phenotypes under those conditions.

The capacity to adjust root mass ratio favoring root growth is expressed most effectively by species that have evolved in fertile soils [55]. Perhaps these sorghum cultivars cannot compete effectively in high-P environments. We wondered if minimum P is required to stimulate root growth. Thus, root growth in sorghum might be dependent on or proportional to P availability, a P pulse hypothesis. Moreover, alteration in root characteristics is only one of the mechanisms plants use to access soil P. For example, the P deficiency in soybean may stimulate root hair elongation rather than lateral root development [57]. Biochemical processes at the soil–root interface may also be more critical in other species [55]. Also, most studies that reported increased root growth under P stress were conducted with seedlings [58], but we cultivated plants under field conditions to maturity. The severity of abiotic stresses under field conditions can vary over time and could be mediated by several uncontrollable variables such as soil moisture, temperature, type, and several others, including the microbial community that affect P dynamics [59]. Field conditions might also give rise to a combination of stresses simultaneously, resulting in nonspecific or conflated responses. Consistent with our results, P deficiency considerably reduced lateral root growth in rice [58] and soybean [57]. In agreement with Parra-Londono et al. [8], we recorded reduced solidity and network area under low-P conditions. As we advance, comparing more ecologically contrasted sorghum populations would be interesting since differentiation in root system traits in response to P availability has been observed previously for more diverse sorghum species [60].

### There was a genotypic effect on root trait plasticity

Here, RDPI for most traits in the white sorghum was highly significant than those in the red sorghum (Fig. 5A). Still, this apparent superiority in plasticity was not reflected in productivity, an occurrence we cannot explain immediately. Even so, phenotypic plasticity could manifest as exploitative or conservative [61]. Species that exhibit exploitative plasticity typically present more considerable phenotypic plasticity to exploit diverse environmental conditions. These species adapt well to local environments to efficiently use the available resources. Conversely, conservative species are characterized by phenotypic constancy that could also be favored in poor nutrient conditions [61]. It appears, at least theoretically, that the white sorghum genotype is more plastic and exploitative. It can make structural and physiological adjustments in its root system and adapt better to local conditions by increased exploitation of the soil environment. Admittedly, these comments are very speculative in the context of the present data, given that the white sorghum had lower shoot biomass in high P and therefore was relatively less productive. On the other hand, the red genotype may have a comparative advantage under less productive conditions where phenotypic stability is more likely to be beneficial than phenotypic plasticity [5,61].

### A larger magnitude of plasticity in root biomass-related traits

We found a high degree of plasticity in root biomass, while the morphological root traits studied presented relatively minor plasticities (Fig. 5). This observation suggests that sorghum’s root biomass might be more dynamic and responsive to local conditions than other morphological root traits. Therefore, extensive root biomass in sorghum will be necessary with decreasing soil P availability. A larger magnitude of plasticity in root biomass-related traits have previously been reported, but these were found in roots of tree species competing with other roots [42,62]. The low plasticity of morphological traits, such as root angle-related and root diameter-related traits, was unexpected. It has been suggested that these traits typically respond to P deprivation to improve P uptake [63]. Phosphorus-efficient bean genotypes, for example, exhibit shallower basal root growth angles and more remarkable basal root angle plasticity in P-deficit conditions [64,65]. Root biomass and root morphological traits may adapt to enhance P uptake and plant performance under P deficiency in sorghum, but the magnitude of adaptation might differ. It is also possible that these mechanisms either act exclusively or complementarily, but this has to be determined.

Furthermore, we hypothesized that the extent of root trait plasticity would depend on the gradient of P availability. Our study presented limited evidence for a difference in root trait plasticity based on the gradient of P availability or the magnitude of the P deprivation. Generally, the RDPI for both genotypes showed a consistent trend for the comparisons between 0 kg P ha^−1^ on one side and 15, 30, 45, 60, and 70 kg P ha^−1^ on the other side (Figs. 5B and 5E), indicating, to a large extent, very similar trait plasticities irrespective of the gradients of P availability. In the present study, most observed changes in root trait values under low P levels compared to high P levels might thus be reflective of just the resource availability and not adaptive to gradients of the resource availability.

### MFAs reveal multidimensional space in sorghum root traits

lant roots have developed several adaptations to help them establish, grow, and function in soils. The many adaptations may have resulted in an inherent dimensionality crucial to the root system’s performance. These dimensionalities might be analogous to the simple building blocks used in describing the RSA, including root geometry, morphology, topology, and growth dynamics [41]. Some of these adaptations in response to environmental conditions could result in trade-offs among traits and give rise to dimensionalities concerning specific stresses. Therefore, we used MFA to summarize several root traits into groups to analyze relationships among the groups. As was the case in this study, MFA is employed when multiple characteristics are measured on the same observations, subject, or element.

Here, the MFA produced 2 independent axes (Figs. 3B to 3D), indicating the existence of multidimensional space in sorghum root traits. The first dimension was dominated by root system geometry and morphology traits (Figs. 3E and 3F), indicating root exploration capacity and root influence on other plant processes. For example, root system geometry traits such as width may present an adaptation for soil resource acquisition. In contrast, morphology traits such as root diameter may influence radial water flow and the cost of construction and maintenance of the root system [41]. The second dimension was dominated by topology traits, providing developmental information on the root system (Figs. 3E and 3F). The present data, therefore, suggest the existence of 2 root trait spectra. The root exploration and influence spectrum represents intercorrelated root traits concerning exploratory root capacity and root influence capacity on other plant processes per unit root system. The root developmental spectrum represents a suite of intercorrelated features that provide developmental information on the root system. It must be noted, however, that these spectra might not be analogous to the economic spectrum of leaf traits, which, compared to root traits, has received greater research attention. The existence of a belowground root economics spectrum comparable to the leaf economics spectrum, which has a trade-off between resource acquisition and conservation, has not been fully established [66]. We may have captured part of this root trait covariation. Still, it would be sweeping to conclude that the spectra here represent the total morphological and physiological traits that explain root trait covariation and trade-offs between resource acquisition and conservation in sorghum species.

Even so, it was also interesting that all the trait groups on the first dimension or spectrum, including attributes for root length, area, volume, and diameter, were automated features that have been more mathematical [29]. On the other hand, the 3 root trait groups of the second dimension or spectrum, including traits for root numbers, angle and weight, were extracted via manual measurements and may be described as biological [29]. Thus, while the 2 root trait spectra in the present study may have functional significance, they may also reflect the mode of extraction of the root features and could be categorized as automated and biological dimensions or spectra. Automated root analysis software generates precise, quick, large-scale, and cumulative phenotyping data that may be combined with related genetic and environmental data to create a comprehensive picture of root development and function. Often, automated extraction of root traits results in the development of massive amounts of data, some of which do not immediately reveal their biological value, posing plant breeders with difficulty integrating these data into breeding programs. Manual extraction of root attributes may be prone to inconsistencies and mistakes and may not give the necessary high throughput. Still, it may allow for smooth incorporation into breeding programs. Our findings demonstrate the advantages of combining and supplementing various techniques. Manual processes may extract multiple dimensions of the RSA, which automated extraction protocols may miss.

### Groups of root traits contribute to genotypic variation in sorghum

Here, root number, angle, diameter, and width measures met the cutoff in the 2 significant dimensions and are therefore considered essential in contributing to the overall variation in this dataset and warrant further investigations (Figs. 4A and 4B). Root number and angle traits were the leading contributors to variation in the significant dimensions (Fig. 4C). The categories of root traits that explained most variations were highly correlated (Fig. 3B), recording RV coefficients between 0.92 and 0.97. The correlation analyses also supported the existence of strong positive correlations among several of the traits (Fig. 3A). Although most of the variation was explained by groups of attributes in the first dimension, groups of features in the second dimension presented a more detailed description of the data, with root angle and root number traits recording relatively higher Lg coefficients of 1.37 and 1.25, respectively. The results also suggest potential trade-offs among groups of root traits. Due to this possible trade-off between groups of features, one group, in theory, could be selected as a proxy for the other. However, this decision must be indexed with the overall function of each category of traits.

In MFA and PCA, variables that do not correlate with any PC typically present minimal contribution to dataset variations and might be removed to simplify the overall analysis. The top contributing variables to the first few PCs provide insights into which variables underlie variations in the dataset and may help with trait selection for downstream analyses. Adu et al. [22] employed a similar approach and reported that a limited number of traits accounted for variability in the RSA of field-grown cowpea. However, that study was based on individual quantitative features, not categories, representing related traits. When dealing with many individual quantitative traits, it might seem that many characteristics are responsible for any observed variation between genotypes. Still, many of these quantitative traits may arise from the same dimension or one building block of the root system.

Nonetheless, selecting variables based on their contribution to PCs requires prudence. A single variable having a more significant size effect and accounting for most of the variance along the first PC might have a high loading and drive the dimension. Furthermore, the signal might be created by a few more essential factors, and a variable could have a higher weight on numerous components. As a result, considering the purpose before picking a variable based on this form of multivariate analysis may be more enlightening. If the goal is to choose features from a multivariate dataset with an immediate outcome, approaches that yield a more interpretable variable importance measure may be preferable.

## Conclusion

Sorghum remains a critical cereal crop for food security as it has a high potential to feed millions of people. While there is an urgent need to understand RSA and root system plasticity to environmental conditions to support crop improvement, little information exists on the RSA and phenotypic plasticity of sorghum in relation to soil P supply. The functional interpretation of root trait plasticity and variability in sorghum would require the knowledge of several root system traits and their role in the plant’s performance. Here, the RSA and phenotypic plasticity of 2 sorghum genotypes were studied to identify traits that contribute to responses to a gradient of soil P. Root system traits were found to contribute to variability and plasticity in response to external P supply. Both genotypes showed a response to external P application, but significant variations were observed between the 2 genotypes with respect to a majority of the root system traits evaluated. The white sorghum seemed better adaptable to a low-P environment as it showed exploitative plasticity. Root system traits such as convex area, surface area, total root length, volume, and root length diameter ranges showed potential for selection for improvements of the genotypes studied. Extensive root biomass seemed important for adapting to low soil P conditions. However, root length seemed to respond in proportion to P supply, showing a fairly balanced partitioning of resources between mass and length. This appears to be unique but requires further studies for validation. Selection of sorghum genotypes for breeding improvements and P-use efficiency can be based on RSA and plasticity of root system traits to P supply. Further studies on sorghum RSA and plasticity in different soil types are recommended to help elucidate the root system traits that contribute most to responses to P supply.

## Data Availability

All data for this study have been included in the paper and are freely available upon request.
